# Hypoxia, cancer metabolism and the therapeutic benefit of targeting lactate/H^+^ symporters

**DOI:** 10.1007/s00109-015-1307-x

**Published:** 2015-06-24

**Authors:** Ibtissam Marchiq, Jacques Pouysségur

**Affiliations:** Institute for Research on Cancer and Aging of Nice (IRCAN), University of Nice Sophia Antipolis, Centre A. Lacassagne, 33 Avenue, 06189 Nice, France; Medical Biology Department (CSM), Centre Scientifique de Monaco, Quai Antoine 1er, Monaco

**Keywords:** Warburg effect, Monocarboxylate Transporters, MCT, BASIGIN, Lactate, Cancer, Therapy

## Abstract

Since Otto Warburg reported the ‘addiction’ of cancer cells to fermentative glycolysis, a metabolic pathway that provides energy and building blocks, thousands of studies have shed new light on the molecular mechanisms contributing to altered cancer metabolism. Hypoxia, through hypoxia-inducible factors (HIFs), in addition to oncogenes activation and loss of tumour suppressors constitute major regulators of not only the “Warburg effect” but also many other metabolic pathways such as glutaminolysis. Enhanced glucose and glutamine catabolism has become a recognised feature of cancer cells, leading to accumulation of metabolites in the tumour microenvironment, which offers growth advantages to tumours. Among these metabolites, lactic acid, besides imposing an acidic stress, is emerging as a key signalling molecule that plays a pivotal role in cancer cell migration, angiogenesis, immune escape and metastasis. Although interest in lactate for cancer development only appeared recently, pharmacological molecules blocking its metabolism are already in phase I/II clinical trials. Here, we review the metabolic pathways generating lactate, and we discuss the rationale for targeting lactic acid transporter complexes for the development of efficient and selective anticancer therapies.

## Introduction

The discovery in the early twentieth century of genes implicated in cancer and the study of mechanisms directly involved in alterations to DNA have dominated the field of cancer research for many decades [1]. However, while this has highlighted, in part, our understanding of processes of malignant transformation, it has become evident that changes in the genome are not sufficient to explain how cancer cells replenish their stock of energy and building blocks to rapidly divide [2]. This realisation has revived interest in understanding cancer cell metabolism and has launched the concept of “cancer metabolic reprogramming,” first described by Otto Warburg a century ago [3]. Several studies over the last decades have shed light on the link between oncogenes, tumour suppressors, metabolic remodelling and tumour growth. However, while these studies confirmed the increased rates of glycolysis of cancer cells, as reported by Warburg, they also show that these cells are addicted to glutaminolysis [4–6]. These two pathways, among others, cooperate to satisfy the demand for ATP, carbon skeletons, and nitrogen required for synthesis of macromolecules of the tumour cells. In addition, increasing evidence supports the role of changes in the microenvironment, including nutrient limitation and oxygen availability, in modulating cancer metabolism. Thus hypoxia, through the hypoxia-inducible factors (HIFs), is considered to be a key player in the transactivation of genes implicated in altered metabolism, leading to the accumulation of diverse metabolites in the microenvironment that promote tumour growth and metastasis [7–9]. Among these metabolites, lactate is drawing the attention of the cancer research community, not as a by product of fermentative glycolysis, but more as a metabolic modulator at the interconnection between different cancer hallmarks including, sustained angiogenesis, evasion of immune surveillance and reprogramming of energy metabolism [10, 11, 1]. Therefore, proteins regulating lactate metabolism offer promising opportunities for developing new anticancer therapies [12–14]. In this review, we will emphasise the metabolic pathways implicated in lactate production, summarise the role of lactate and lactate transporters in tumour development and highlight the recent advances, benefits and risks of future therapies based on inhibition of lactate transport.

## Remodelling of cancer metabolism: an efficient way to maintain cellular bioenergetics and macromolecular biosynthesis

### Glucose metabolism

In the 1920s, a German scientist, Otto Warburg, reported abnormalities in cancer cell metabolism, which opened the door to a new large field of cancer studies. He demonstrated that unlike the majority of normal cells, which rely primarily on mitochondrial oxidative phosphorylation (OXPHOS) to produce energy, tumour cells ardently take up glucose to perform aerobic glycolysis [15, 3]. This phenomenon, referred to as Warburg effect, became a distinctive metabolic characteristic of cancer cells, and proved useful for clinical detection and monitoring of tumours by [^18^F]-deoxyglucose positron emission tomography (FDG-PET) imaging [16, 17]. Initially, the Warburg effect was proposed to be a result of impairment of mitochondrial respiration [3]. However, numerous recent studies have shown that mitochondrial OXPHOS in many tumours is intact [18, 5] Instead, the Warburg effect is proposed to be due to increased glycolysis that suppresses OXPHOS, which is caused by adaptation to hypoxic conditions at the early avascular stages of tumour development [19, 20].

Since the ATP yield of aerobic glycolysis (2 ATP per glucose molecule) is 18-fold lower than that of OXPHOS (36 ATP per glucose molecule), metabolic reprogramming implicates an increased rate of glucose uptake by tumour cells to meet the energy, macromolecular biosynthesis and redox needs required for rapid proliferation [21, 22]. Thus glucose transporters and downstream glycolytic enzymes are overexpressed in more than 70 % of cancers [23, 24]. This up-regulation is mainly driven by the hypoxia-induced transcriptional factor HIF-1 and by Myc, alone or in cooperation. Additional factors exacerbating growth and metabolism include oncogenes (Akt, PI3K, mTOR, Ras, Raf) and loss of tumour suppressor genes (VHL, PTEN, p53) [22, 25]. Oncogenes and tumour suppressors are also critical activators of HIF-1α [26, 27], leading to increased translation (PI3K, PTEN) and stabilisation (VHL) of HIFs in an O_2_-independent manner. Consequently, the transcription of a wide range of genes occurs, some of which are implicated in metabolic reprogramming [28, 8]. Elevated HIF-1α levels in rapidly growing cells, like embryo and tumours, not only stimulates glycolysis but restricts mitochondrial respiration through the inhibition of the mitochondrial pyruvate dehydrogenase (PDH), reducing pyruvate flux into the tricarboxylic acid (TCA) cycle [29, 30]. This HIF-1-mediated inhibition of PDH in reprogramming glucose flux is a major basis of the Warburg effect.

**Fig. 1:**
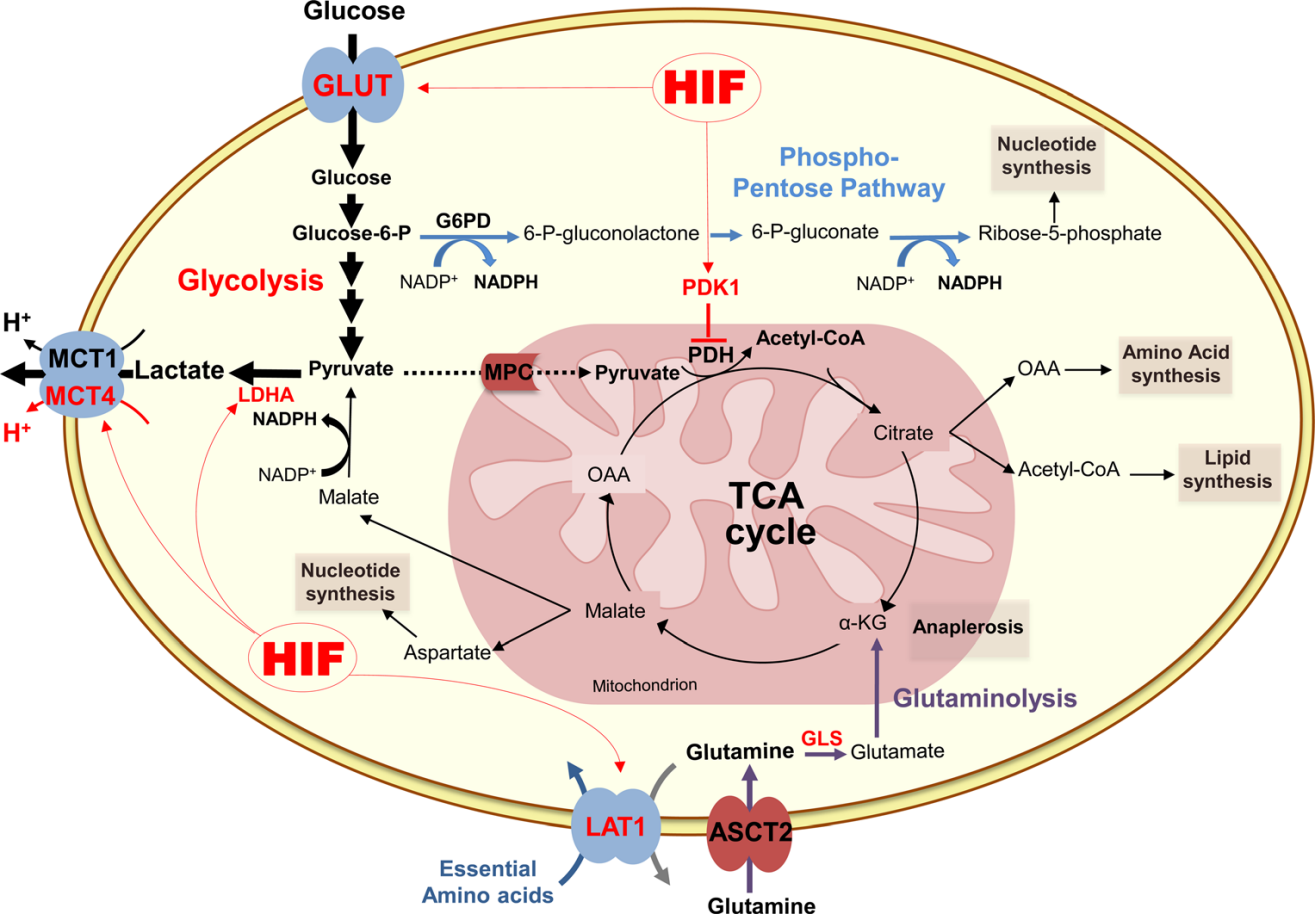
Schematic representation of glucose and glutamine metabolism in cancer cells. After entering the cell through specific transporters (GLUT), glucose is metabolised to pyruvate. In cancer cells, pyruvate is mainly converted to lactate by the lactate dehydrogenase A (LDHA), while its catabolism in the tricarboxylic acid (TCA) cycle is restricted through the inhibition of the mitochondrial pyruvate dehydrogenase (PDH) by the pyruvate dehydrogenase kinase 1 (PDK1) induced by HIF-1. Glycolysis (*bold arrows*) generates also another important intermediate, glucose-6-phosphate (glucose-6-P) that is metabolised by the pentose phosphate pathway (*blue arrows*), which produces NADPH and ribose-5-phosphate for glutathion and nucleic acids synthesis. Glutaminolysis (*purple arrows*) is an alternative energy source for cancer cells. First converted to glutamate by glutaminase (GLS) in the cytosol, glutamine replenishes tricarboxylic acid (TCA) cycle (anaplerosis) through the conversion of glutamate to α-ketoglutarate (α-KG). Glutaminolysis contributes also to synthesis of lipids, amino acids, nucleotides and generation of lactate that is transported out of the cell by the ubiquitous monocarboxylate transporter 1 (MCT1) and the hypoxia inducible MCT4. Hypoxia-inducible factor (HIF), glucose-6-phosphate dehydrogenase (G6PD), mitochondrial pyruvate carrier (MPC), oxaloacetate (OAA), l-type amino acid transporter 1 (LAT1), Asc-type amino acid transporter 2 (ASCT2)

In most cancer cells, glucose is not only used to perform glycolysis but can also be metabolised by alternative pathways such as the pentose phosphate pathway (PPP) (Fig. 1). By generating NADPH and ribulose-5-phosphate, PPP promotes glutathione production, fatty acid, sterol, and nucleic acid synthesis, which helps cells to counteract oxidative stress, facilitates DNA damage repair and confers resistance to chemotherapy and radiation [31, 32]. Thus, to meet their constant demand of nucleotides and biosynthetic precursors, malignant and proliferative tumours frequently up-regulate the PPP via different mechanisms [33–36].

### Glutaminolysis

Alongside glucose metabolism, many studies highlight the crucial role of glutaminolysis in tumour cell bioenergetics and metabolism. It was demonstrated that glutamine consumption is substantially increased in many cancers compared to other amino acids, and represents a feature of malignancy [6, 37, 38]. Indeed, glutamine is a key nutrient for several anabolic and catabolic processes leading to ATP generation, redox homeostasis, TCA cycle replenishment (anaplerosis), intracellular antioxidant pool maintenance and macromolecular synthesis [4, 39, 40] (Fig. 1). As for other metabolic pathways in cancer, up-regulation of glutaminolysis is positively driven by oncogenic signals. The best-characterised regulatory mechanism implicates the transcription factor c-Myc [41–43] and a variant of the Rho GTPases family, the oncogenic diffuse B cell lymphoma protein (Dbl) [44]. In addition, recent reports have shown that loss of the retinoblastoma tumour suppressor, as well as KRas and HIF activation, promote glutamine utilisation and metabolism in cancer cells [45–48].

In summary, during tumour development, cells undergo metabolic reprogramming due to a combination of a poor and leaky vasculature, hypoxia and oncogenic signalling. Interaction between aberrant metabolic pathways provides cells not only with energy and macromolecules, but also a modified microenvironment. Thus, through the production of diverse metabolites and enhanced glucose and glutamine metabolism, a perfect nest is created for tumour cell growth and survival.

## Lactate: a key metabolic modulator of cancer cells and stroma

### Lactate, hypoxia and acidosis

Lactate production, tumour acidosis and hypoxia are commonly thought to be linked. However, many studies have shown that high lactate concentrations are not necessarily associated with hypoxia and that the two phenomena occur at different sites throughout tumours [49, 50]. Aerobic glycolysis, increased glutaminolysis and low perfusion rates of blood vessels may also contribute to lactate accumulation in non-hypoxic areas [15, 25, 51]. Moreover, when considered separately, the clinical significance of the two concepts is also distinct; while hypoxia without lactic acidosis is usually associated with poor prognosis, lactic acidosis in the absence of hypoxia has been recently shown to shift energy utilisation of breast cancer cells from glycolysis towards OXPHOS, contributing thus to favourable clinical outcomes [52]. Moreover, the high conversion rate of pyruvate into lactate, via the enzymatic activity of lactate dehydrogenase A (LDHA), is usually assumed to be the major mechanism responsible for tumour acidity. Nevertheless, using Ras-transfected Chinese hamster lung fibroblasts, Newell et al. [53] showed that glycolysis-deficient cells have similar extracellular pH (pHe) values than the ones of parental cells, in both in vitro and in vivo, even if the former produced less lactate. They suggested, therefore, that lactic acid accumulation resulting from enhanced glycolysis is not the only process that generates tumour acidosis. Similar results were reported by two different studies using LDH-deficient cells [54] and glycolysis-impaired cell lines [55], which found that besides lactate, increased levels of CO_2_ generated by oxidative metabolism were the main cause of tumour acidity. In fact, both the TCA cycle and the PPP produce CO_2_ that is hydrated by carbonic anhydrases (CA) to generate HCO_3_^−^ and H^+^ [56, 25]. To overcome intracellular acidification (pHi), cells developed adaptive strategies to extrude acid; including H^+^ export and HCO_3_^−^ import [57–59]. Collaboration of these mechanisms leads to the acidification of the tumour microenvironment and decreased pHe, creating a reversed pH gradient (pHe (6.6–6.9) < pHi (7.2–.5)) that supports the malignant phenotype [60, 1, 61].

**Fig. 2:**
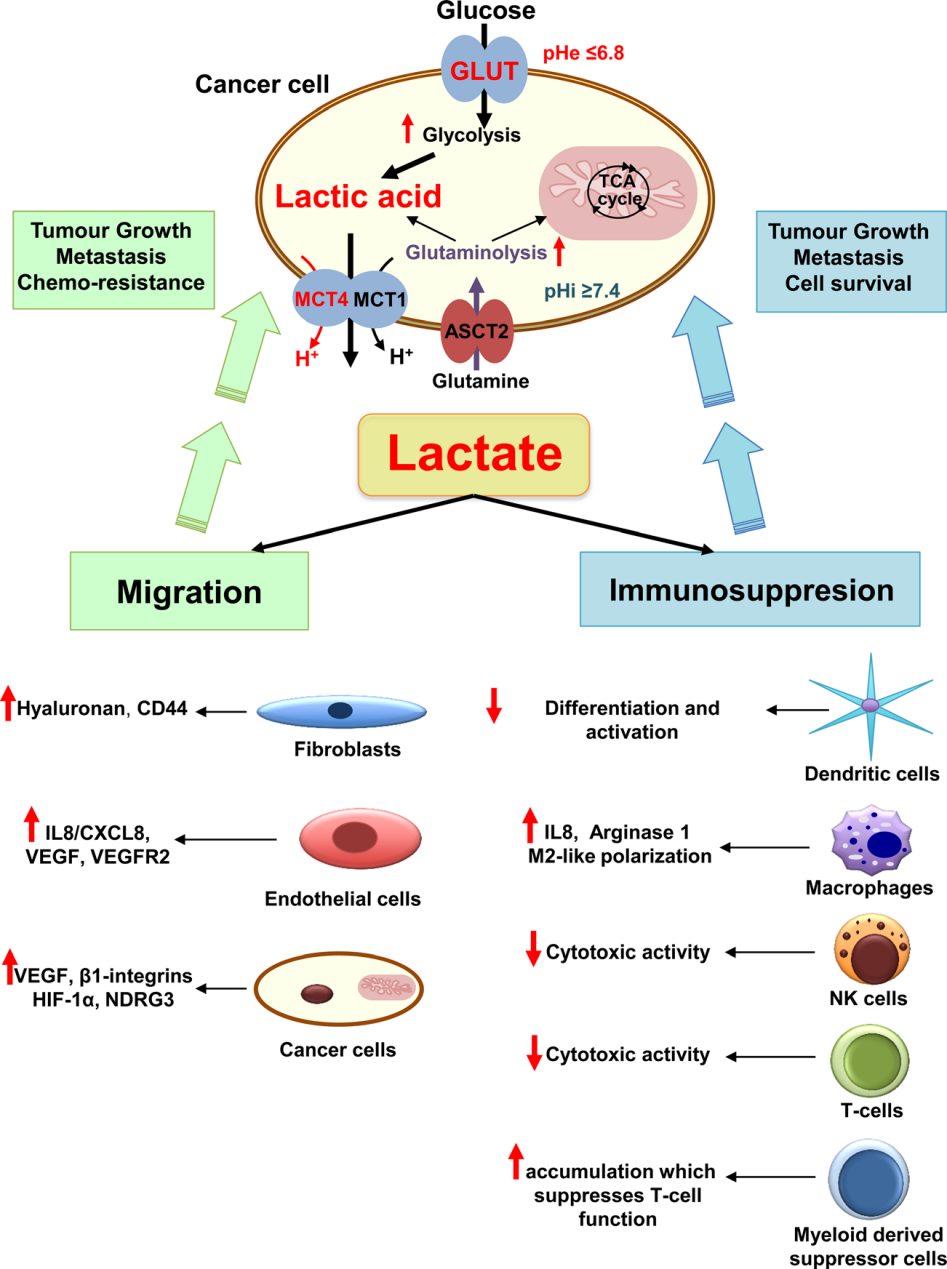
The different roles of cancer-generated lactic acid in promoting tumour growth and metastasis. Enhanced glycolysis and glutaminolysis generate large amounts of lactic acid that is exported by monocarboxylate transporters (MCT) 1 and 4. The accumulation of lactic acid in the extracellular milieu induces drop in extracellular pH (pHe), acidification of tumour microenvironment and promotes several cancer processes leading to cell survival, tumour growth and metastasis. Lactic acid stimulates angiogenesis by increasing the production of the vascular endothelial growth factor (VEGF) and its receptor VEGFR2 by tumour and endothelial cells. Lactate drives also angiogenesis through the activation of hypoxia-inducible factor 1 (HIF-1), N-Myc downstream-regulated gene 3 (NDRG3) protein and the stimulation of the production of interleukin 8 (IL8). Increased extracellular lactate levels influence cancer cell motility by promoting hyaluronan production, which acts on fibroblasts and cancer cell cytoskeleton through interaction with CD44. More importantly, lactate generated by altered cancer metabolism plays an important role in escape of immune surveillance, mostly through decreased cytotoxic activity of human T lymphocytes (T cells) [75, 76] and natural killer (NK) cells. Further, lactate reduces dendritic cell maturation, induces the accumulation of myeloid derived suppressor cells, and promotes M2-like polarisation of tumour-associated macrophages

Although lactic acid is not the major player in pHe acidification of cancer cells, an increasing number of recent studies underline its important role as a “signalling molecule” involved in different mechanisms promoting cancer cell survival, proliferation and metastasis [62, 10] (Fig. 2).

### Lactate a signalling molecule of cell migration and angiogenesis

The lactate released by cancer cells is noticeably recognised as an angiogenic promoter. Several recent studies indicate that lactate increases the production of the vascular endothelial growth factor (VEGF) and its receptor VEGFR2 by tumour cells and endothelial cells, respectively. This induction occurs through the activation of HIF-1 as the result of an indirect accumulation of pyruvate, an inhibitor of HIF-prolyl-hydroxylases (PHDs) [63–65, 14, 66]. However, impact of lactate on angiogenesis is dependent not only on HIF-1 expression. Vegran et al. have also shown that lactate could induce interleukin-8 (IL-8) production by endothelial cells, through nuclear factor-kappa B (NF-kB) stimulation, which resulted in new blood vessel maturation and increased cell migration [67, 68]. Furthermore, a recent study reported a direct role of lactate in modulating angiogenesis independently of HIF. Thus, through the stabilisation of N-Myc downstream-regulated gene 3 (NDRG3) protein expression, lactate induced up-regulation of VEGF, IL-8 and CD31 levels during prolonged hypoxia, apparently via the ERK1/2 signalling pathway [69]. Alterations in extracellular lactate levels have also been shown to increase the in vitro random migration of different cancer cells in a concentration-dependent manner, which could facilitate metastasis [70, 10]. Furthermore, lactate induced cancer cell motility by increasing production of other factors such as transforming growth factor-β2 (TGF-β2), hyaluronan and CD44, which play an important role in integrin activation, angiogenesis, stemness and modulation of stroma [71, 70, 72–74] (Fig. 2).

### Immuno-modulatory role of lactate

Besides promoting angiogenesis and migration, the metastatic potential conferred by lactic acid is linked to its emerging role in escape of immune surveillance [62]. Cancer-generated lactic acid was described to strongly inhibit the anticancer immune response through a decrease in the cytotoxic activity of human T lymphocytes [75, 76] and natural killer cells [77, 78]. Lactate was also reported to inactivate cytokine release from dendritic cells and to inhibit the differentiation and activation of monocyte-derived dendritic cells [79–81]. Enhanced immune suppression by lactate was further linked to its role in inducing the accumulation of myeloid derived suppressor cells, which further suppresses the T lymphocytes’ function [77]. Other studies have shown that lactate also promoted tumour-associated inflammation by increasing the production of cytokines such as IL-23 and IL-6 [62, 82]. Moreover, recent data from syngeneic murine tumour models of Lewis lung carcinoma (LLC) and B16-F1 (B16) melanoma cancer cell lines showed that lactate-induced stabilisation of HIF-1α increased arginase 1 expression and consequently M2-like polarisation of tumour-associated macrophages [83] (Fig. 2).

In view of the above, a critical role has been attributed to lactate in the development and progression of a wide range of cancers, leading to consider it as a relevant prognostic marker of poor patient survival. To confirm this, data from Mueller-Klieser’s group showed for the first time that lactate accumulation was tightly linked to primary cervical cancer aggressiveness and therefore inversely correlated with patient survival [84, 85]. This negative correlation was also confirmed for patients with head and neck squamous carcinoma (HNSCC) pre-treated with lactate [11, 86]. Another extensive and independent study have shown that lactate but not pyruvate concentration correlates significantly with tumour response to fractionated irradiation in tumour xenografts of 10 human HNSCC cell lines [87]. Furthermore, high lactate levels were described, as a potential marker of human rectal adenocarcinoma [11], glioblastoma [88, 89] and prostate tumour aggressiveness [90, 91], as far as it is positively associated with resistance to radiation and probability of metastasis.

## Lactic acid transporter complexes: structure, expression and regulation

To achieve activation of cell motility and suppression of the immune system, as described above, cancer cells have to maintain a continuous flux of glycolysis that is intimately linked to the rate of lactic acid extrusion. For many years, lactate was thought to be removed from cells merely via transmembrane diffusion of its undissociated form, lactic acid. However, studies from Halestrap’s group on human red blood cells established the presence of specific transmembrane lactate transporters, belonging to a family of monocarboxylate transporters (MCTs) [92, 93]. This family includes 14 members coded by the *Solute Carrier family 16* (*SLC16A*) gene, which show sequence homology [94]. However, only the first four isoforms (MCT1-4) have been functionally validated to transport monocarboxylates, such as l-lactate, pyruvate and ketone bodies [94, 92]. This transport is mainly controlled by the H^+^ and monocarboxylate concentration gradient across the plasma membrane, which determines the net direction of transport (influx or efflux) [95]. MCTs display distinct substrate affinity and tissue distribution, and play an important metabolic role in many physiological and pathological situations, extensively reviewed by Halestrap [92, 96]. For concision and clarity, we will focus only on the structure and function of the well-characterised proton coupled MCT1 and MCT4.

### MCT1/MCT4

Due to the well-established role of lactate in metabolism and pH homeostasis within many tissues such as muscle, brain, kidney, liver and retina, a large number of studies concern the lactate/H^+^ symporters MCT1-4. Although the crystal structure of MCTs has not been described, topology predictions indicated that these proteins contain 12 transmembrane domains (TMs) with intracellular N- and C-termini and a large cytoplasmic loop connecting TM6 and TM7. This prediction was later confirmed for rat MCT1 [94, 92, 97].

The likely transport mechanism of both H^+^ and lactate in MCTs was first identified for MCT1 and suggested a translocation cycle including “outside-open” and “inside-open” conformations [98–100] implying interactions between lactate, H^+^ and MCT1 key residues (K38, D302 and R306) [94, 101, 102]. This mechanism is assumed to be shared by the other three MCTs (MCT2-4), as sequence alignment shows that 70 % of the transmembrane amino acids are highly conserved for human MCT1-4. Thus, point mutations within the transmembrane domains of these transporters have been demonstrated to affect their substrate specificity, transport activity and inhibitor sensitivity [103, 104]. Indeed, even if MCT1-4 share common features and transport the same substrates, they show different binding affinities for monocarboxylates. Therefore, MCT1 and MCT2 transport pyruvate (*K*m ≈ 0.1–0.74 mmol/L) and stereoselectively l-lactate (*K*m ≈ 1–3.5 mmol/L) with a very high affinity [94, 95], compared to MCT4 which posses a lower affinity for both pyruvate (*K*m ≈ 153 mmol/L) and lactate (*K*m ≈ 28 mmol/L) [105, 106]. Consequently, the heterogeneous affinities correlate with different expression patterns within tissues [107]. MCT4, due to its very high *Km* for pyruvate and lactate, is mainly expressed in highly glycolytic cells such as white skeletal muscle fibres and astrocytes, while either or both MCT1 and MCT2 are expressed in red skeletal muscle, heart and neurons where they uptake lactate to fuel OXPHOS. MCT3, however, is exclusively expressed on choroid plexus and the basolateral membranes of the retinal pigment epithelium [108], and was shown to transport l-lactate with a *Km* of 6 mmol/L.

Differences in tissue distribution imply necessarily distinct regulatory mechanisms. Thus, while little is known about the regulation of MCT2 and MCT3 expression, different studies highlighted the regulation of both MCT1 and MCT4 expression. Analysis of the 5ʹ-UTR region of these two MCTs suggests that both transcripts may undergo distinct transcriptional and post-transcriptional regulatory mechanisms. Indeed, MCT4 expression is up-regulated in hypoxia through HIF-1 binding to two hypoxia response elements (HRE) upstream of the transcription start site [109]. However, while there is no evidence of a HRE on the MCT1 gene sequence, the MCT1 promoter contains potential binding sites for a number of other transcriptional factors, such as MYC, PGC-1α, NRF-2 and CREB [13, 110]. Direct interaction between the p53 and MCT1 gene promoters was recently described by Ferron’s group and resulted in altered MCT1 messenger RNA (mRNA) stabilisation in hypoxia [111]. MCT1 expression can also be regulated in muscle cells after intense exercise through accumulation of lactate and activation of calcineurin and AMP-activated protein kinase (AMPK) [112, 94, 110]. Further, in the pancreatic insulin secreting β cells, MCT1 is regulated by either epigenetic modification within CpG islands or microRNA-29, which target the 3ʹ-UTR region inducing MCT1 mRNA degradation and translational repression [113, 114]. Substances such as butyrate [115, 116], testosterone [117] and thyroid hormone T3 [118] have also been described to stimulate MCT1 tissue expression.

### CD147/*BASIGIN*

**Fig. 3:**
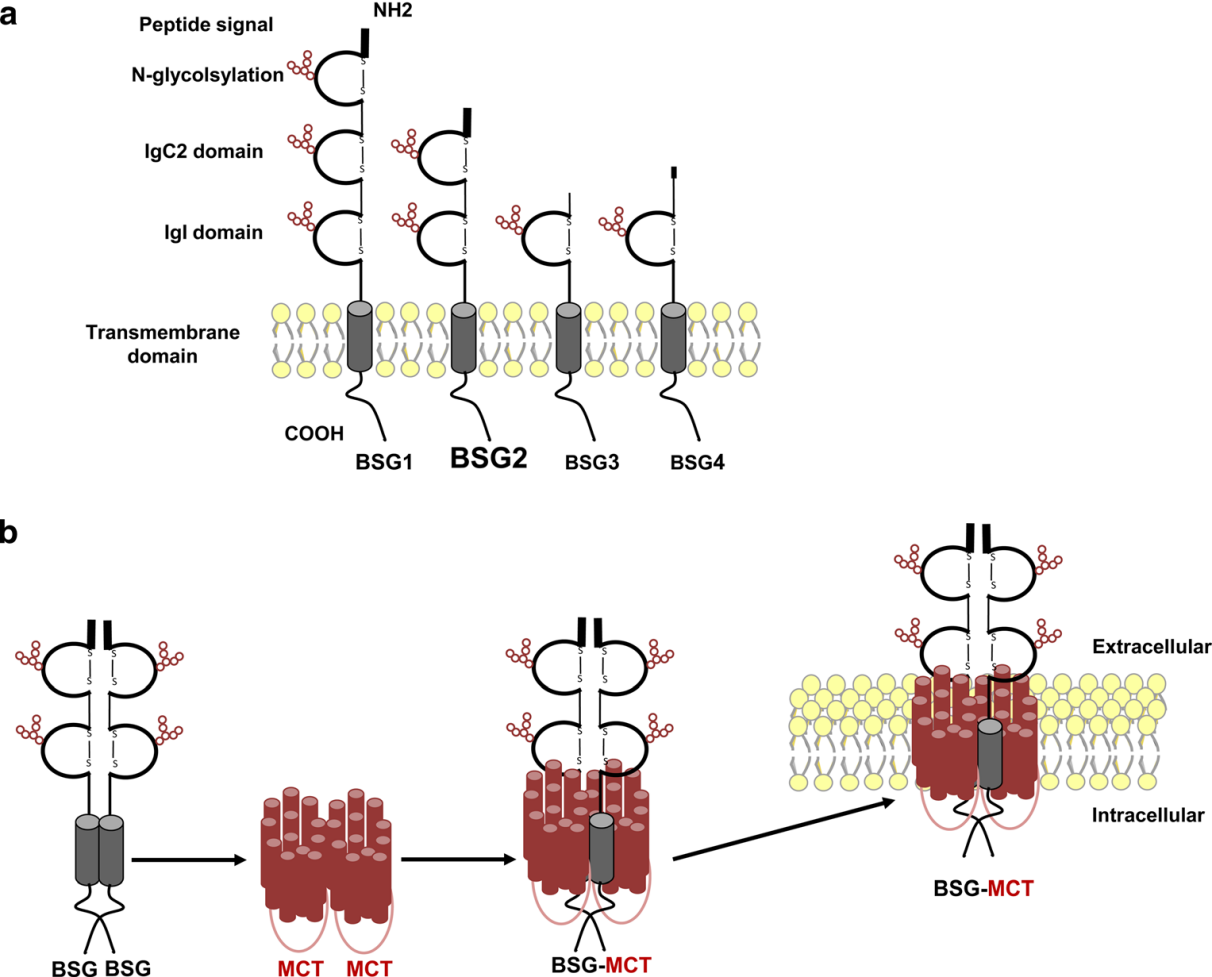
Schematic representation of BASIGIN (BSG) isoforms structure and interaction with monocarboxylate transporters (MCT). **a** Alternative transcriptional initiation and variation in splicing results in four isoforms of BSG (BSG1-4) that are composed of extracellular Ig-like domains containing glycosylation sites (*red circles*), a single-membrane spanning segment and a short intracellular cytoplasmic tail. BSG1 is specifically located at the retina, BSG2 (*in bold*) is the most prevalent isoform and BSG3/BSG4 are intracellular, lacking signal peptide and much less abundant proteins. **b** Dimer of BSG binds to two monomers of MCT, illustrated by 12 individual helices each, and forms a homo-oligodimer that translocate to the plasma membrane for proper expression and functionality

Besides genetic regulation of MCT1-4, as described above, many studies published in the early 2000s have shown that these non-glycosylated plasma membrane transporters require a tight association with transmembrane glycoproteins for proper folding and trafficking to the cell surface. Using co-immunoprecipitation and chemical cross-linking, Kirk et al. showed that MCT1 and MCT4 specifically interacted with CD147/BASIGIN (BSG) [119]. BSG (also named EMMPRIN, gp42, HT7, neurothelin, 5A11, OX-47 and M6) is a transmembrane glycoprotein of the immunoglobulin (Ig) superfamily composed of extracellular Ig-like domains, a single-membrane spanning segment and a short intracellular cytoplasmic tail [120]. Alternative transcriptional initiation and variation in splicing results in four isoforms of BSG (BSG1-4) [121] (Fig. 3a).

While little is known about the expression and functionality of both BSG3 and BSG4, studies have shown that the BSG1 isoform (three Ig-like domains) is specifically located at the retina where it closely interacts with MCT3 [122, 123]. However, BSG2 (referred to as BSG from now on) is the most prevalent and studied isoform. It contains two Ig-like domains and is widely expressed in tissues where it interacts with MCT1 or MCT4 [120]. Cross-linking experiments together with studies using fluorescence resonance energy transfer revealed that BSG forms a homo-oligodimer in which a dimer of BSG binds to two monomers of MCT1 [124] (Fig. 3b).

To reach the plasma membrane, MCT2 is also assisted by two paralogs of BSG, a developmentally expressed protein named EMBIGIN (gp70, EMB) [94, 125] and synaptic glycoproteins called neuroplastins (Np55 and Np65) [126].

Many in vitro studies have demonstrated tight collaboration of MCTs and BSG for correct plasma membrane expression. Although the first experiments showed that BSG co-localised with MCT1 on the cell surface and that co-transfection with BSG complementary DNA (cDNA) facilitated the expression of MCTs on the plasma membrane, we and others have recently shown that the surface expression and trafficking of BSG is also dependent on its association with MCTs. Thus, knockdown or gene disruption with zinc finger nucleases (ZFN) of MCT4 alone, or in combination with MCT1 knockdown, impaired the maturation of BSG, leading to its accumulation in the endoplasmic reticulum and proteasomal destruction [127, 128, 14].

This dependency was also emphasised in in vivo studies of *BSG*-*null* mice, which showed that *BSG* gene knockout resulted in a substantial reduction in the immunohistochemical staining intensity for MCT1 and disrupted its distribution in almost all tissues [129, 130]. BSG is involved in many physiological events, such as spermatogenesis, implantation, fertilisation, lymphocyte responsiveness, vision, behaviour and memory [120, 131]. Considering the dependence on bioenergetics of all these events, the in vitro and in vivo studies mentioned above are consistent with a direct impact of a decrease in MCT expression in the phenotype of BSG-null mice (blindness, sterility, immunodeficiency, and problems with learning and memory) [132, 133, 120, 129].

However, the question whether BSG is the only ancillary protein of MCT1, 3 and 4 remains to be answered. Indeed, MCT1 has been shown in some tissue to be properly expressed independently of BSGs [129]. We have also recently reported functional residual MCT1 and MCT4 expression in different *BSG*-null cancer cell lines [134, 14], suggesting the presence of unidentified proteins or mechanisms for targeting these MCTs to the cell surface. Experiments employing co-immunoprecipitation and chemical inhibition point to a role of CD44, a receptor for hyaluronan, as a co-chaperone of MCTs [135]. Further investigation of CD44 expression in *BSG*-null mice and cancer cell lines should be performed to understand the implication of this receptor in lactate transport.

## Clinical significance of MCTs and BSG in cancer

### MCTs in cancer

**Fig. 4:**
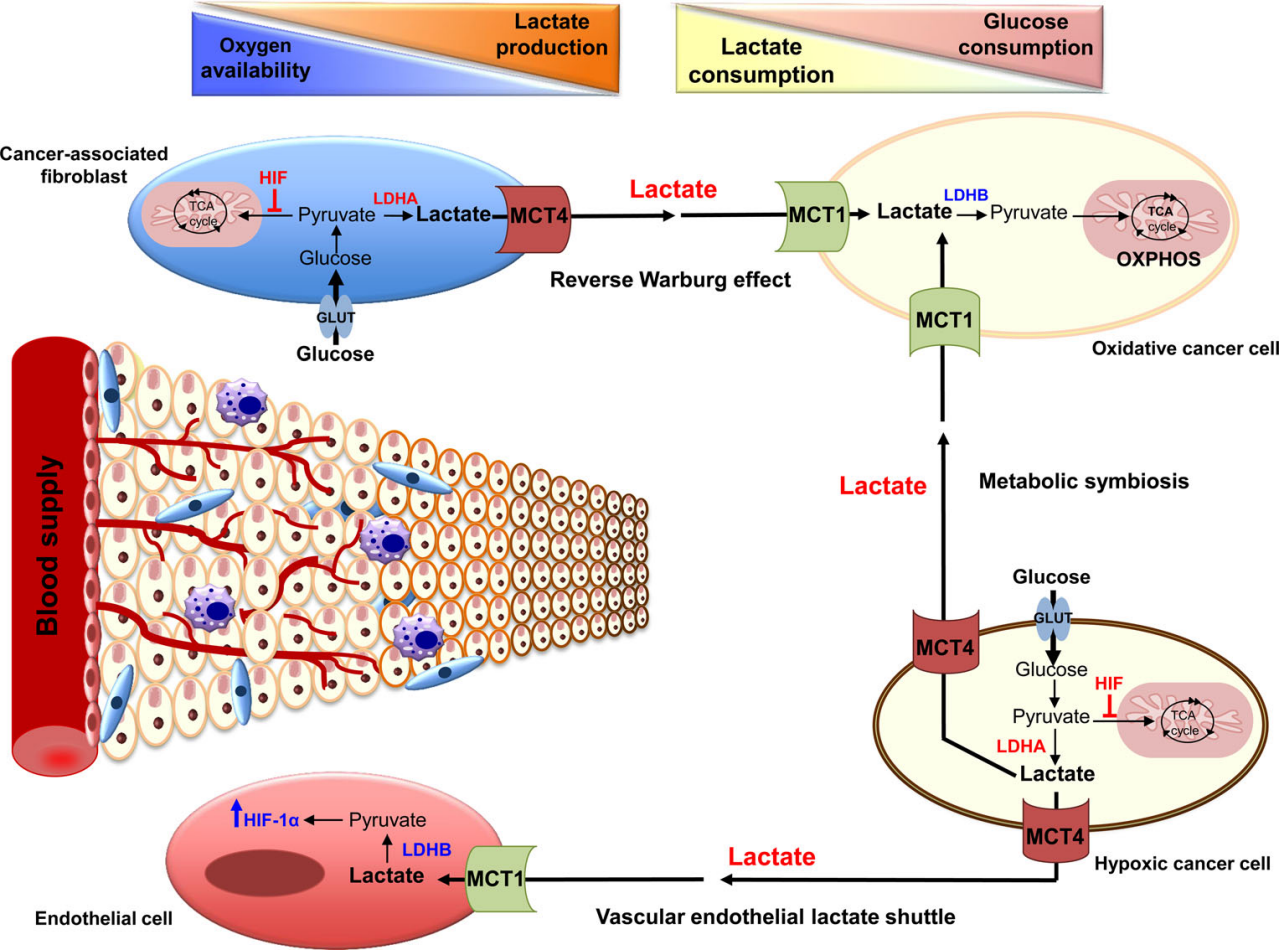
Model of tumour microenvironment and lactate shuttles in cancer. Cells located far from the perfused blood vessels become rapidly hypoxic and rely on glycolysis for proliferation. They generate, therefore, large amount of lactic acid that is extruded by monocarboxylate transporter 4 (MCT4). Lactate is subsequently taken up by the endothelial cells via monocarboxylate transporter 1 (MCT1), and is converted into pyruvate by the lactate dehydrogenase B (LDHB), a phenomenon referred to as “vascular endothelial lactate shuttle.” Pyruvate, by stabilising hypoxia-inducible factor 1 α (HIF-1α), induces tumour angiogenesis. Normoxic cancer cells, that highly express MCT1, also preferentially take up lactate produced by hypoxic cancer cells to perform oxidative phosphorylation (OXPHOS). This “metabolic symbiosis” allows hypoxic regions of the tumour to acquire high levels of glucose and, subsequently, generate lactic acid. In addition, cancer-associated fibroblasts, which are highly glycolytic and express MCT4, also supply oxidative cancer cells with lactate. This tumour-stroma cooperation, termed “reverse Warburg effect” in addition to the other lactate shuttles, result in the establishment of lactate and glucose consumption gradients within tumours

Since many cancer cells rely primarily on glycolytic metabolism to support rapid proliferation, they produce increased amounts of lactic acid that should be efficiently extruded from cells to the tumour microenvironment for cell survival. Thus, up-regulation of MCT1 and MCT4 has been reported in several solid tumours, such as glioblastoma, breast, colon, liver, ovarian and lung cancers [136]. However, the distribution pattern of these two MCTs is different due to the disparities in the lactate content and its utilisation between tumour types, the oncogenic pathways driving each cancer and the distinct regulatory mechanisms of each MCT. MCT1/MCT4 expression was also shown to differ even within the same tumour. Sonveaux et al. [137] have shown that the well-oxygenated tissues of human cervical and colon xenograft tumours express high levels of MCT1 compared to almost no detectable expression in hypoxic regions. Although an interesting inter-tumour coupling model, this notion has remained highly controversial and not observed in other tumour types. Other studies have also reported increased expression of MCT4, along with other glycolytic proteins in hypoxic and poorly vascularised tumour regions [138, 139]. These differences are consistent with the notion of metabolic cooperation or “micro-ecosystem” as recently described by different groups. This implies that different tumour cell populations reprogram their metabolism and implicate complementary pathways to meet the challenge of energy production and macromolecule synthesis in a nutrient-limited environment [140, 137]. Thus, as defined for skeletal muscle, brain and liver, a lactate shuttle is established between hypoxic and oxygenated cancer cell populations (Fig. 4). In this model, the lactate that is released as a waste product by hypoxic cells, mainly via MCT4, is taken up and re-used by cells expressing MCT1 in oxygenated regions. Aerobic cells will then convert lactate into pyruvate to fuel their oxidative metabolism, allowing glucose to reach the hypoxic cells at the poorly vascularised tumour core [137]. Thus, using metabolic imaging techniques, Galie et al. [141] validated increased glucose uptake in the hypoxic tumour regions compared to the well-vascularized regions at the periphery of the tumour. Moreover, inhibition of MCT1 in different cancer cells has been reported to decrease lactate availability for oxidative cells, forcing them to take up glucose. This resulted in glucose starvation and cell death of hypoxic cells, leading subsequently to tumour growth arrest [137].

Aberrant metabolism of cancer cells is not alone in promoting the malignant phenotype, components of the tumour microenvironment, such as cancer-associated fibroblasts (CAFs), endothelial cells and inflammatory cells, also play an important role. In addition to the role of lactate in inducing angiogenesis or the so-called vascular endothelial lactate shuttle (Fig. 4), as mentioned above, an increasing number of studies support the existence of tumour-stroma metabolic cooperation. Immunohistochemical analysis of human colorectal adenocarcinomas showed that tumour cells express high levels of GLUT1, MCT1, and HIF-1α, indicating increased anaerobic metabolism and lactate production, whereas the expression profile of CAFs (low level of GLUT1/HIF-1α, and high MCT1/MCT2) suggested high lactate absorption and oxidative metabolism [142].

Recently, increasing evidence supports the presence of another type of tumour-stroma collaboration, referred to as the “reverse Warburg effect” [143–145] (Fig. 4). In this case, CAFs first exhibit a highly glycolytic phenotype and consequently express high levels of MCT4, which is necessary to extrude lactate. This process is proposed to be induced by oxidative stress in association with loss of caveolin-1 and other metabolic alterations. Formerly, epithelial cancer cells use MCT1 to import secreted lactate, which enters the TCA cycle and drives oxidative metabolism. This phenomenon has been described for breast cancer [145], prostate cancer [146], head and neck tumours [147] and osteosarcoma [148]. Further, elevated levels of stromal MCT4 expression were reported to be a marker of poor survival in triple negative breast cancer [145]. However, these studies remain highly controversial as a recent study from our group showed elevated expression levels of glycolytic markers, such as CAIX, LDHA and MCT4, in all breast cancers, with highest rates in triple negative breast cancer. More importantly, staining of tumours for MCT4 but not of the stroma correlated with negative prognostic index for overall-survival [149]. Moreover, recent studies have shown decreased expression or even absence of the LDHB subunit, the enzyme converting lactate into pyruvate, in breast, prostate and gastric cancers due to hyper-methylation of the LDHB promoter [150]. Thus, these recent findings, together with the prominent expression pattern of MCT4 in many cancers raises doubt concerning the reverse Warburg model proposed by Lisanti’s group [145].

### BSG in cancer

In parallel to MCT1/4 overexpression, CD147/BSG is also commonly up-regulated in cancers, and since 1990, more than 540 research articles have highlighted its pro-tumour role. Indeed, deregulation of BSG has been linked to almost every type of cancer [151, 152]. An immunohistochemical study of a large number of normal and cancer tissues has shown that BSG is overexpressed in 112 out of 129 tumour samples with a very high incidence in glioblastoma, breast cancer, pancreatic cancer, hepatocellular carcinoma and squamous cell carcinomas, among others [152]. BSG expression correlated with the histological type of tumours, grade of cancers, tumour progression and recurrence and patient survival. Moreover, BSG expression usually co-localised with MCT1/MCT4 in tumour tissues, which constitutes a prognostic marker of poor clinical outcome [153, 154].

BSG was first named “EMMPRIN” for Extracellular Matrix MetalloPRotease INducer, because it was reported to be associated with increased matrix metalloproteases (MMPs) through which it promoted tumour invasiveness and metastasis. This function, based on nearly 200 studies showing a positive correlation between knockdown or ectopic expression of BSG and levels of different MMPs (1, 2, 3, 9, 11, 14 and 15), was proposed to be mediated via up-regulation of MMPs produced by fibroblasts neighbouring tumour cells [131]. This pro-tumoural model placed BSG at the centre of tumour invasion but so far remains only correlative with no molecular mechanisms demonstrating how BSG or its soluble form is capable of inducing MMPs. Moreover, most of these studies ignored the major role of BSG in tumour metabolism, particularly the control of the glycolytic rate, lactate transport and pHi homeostasis through the assistance of BSG in bringing MCTs to the plasma membrane. Indeed, we and others have shown that targeting BSG with shRNA or deleting the *BSG* gene with zinc fingers nucleases (ZFNs) reduced levels of expression of MCT1/MCT4, increased the intracellular pool of lactic acid and impaired tumour growth in vivo [155, 134, 128, 14, 156].

Recent studies from our group showed that BSG knockout in colon, glioma, and lung cancer cell lines promoted tumour proliferation through metabolic reprogramming [134, 14], but without any significant change in the expression levels of MMPs compared to parental cells. Using co-cultures of either human fibroblasts or mouse embryonic fibroblasts (MEFs) and tumour cell lines we showed, in contrast to the published literature, that the disruption of BSG in tumour cells and in MEFs does not modify the production of MMPs. These studies concerned MMP1 and MMP13, stromelysins MMP3 and MMP11, the membrane type (MT) 1-MMP, MMP14, and finally, the most described gelatinases A and B MMP2 and MMP9 [157].

Besides MCTs and MMPs, BSG was reported to interact with a number of other cell surface regulatory proteins, such as β1-integrins, cyclophilin A, ubiquitin C, caveolin-1, the CD44 glycoprotein, CD98 heavy chain (CD98hc), large neutral amino transporter 1 (LAT1), Asc-type amino acid transporter 2 (ASCT2) and VEGFR2 [158–160, 135, 161, 162, 131, 163]. Interaction with these molecules results in different roles of BSG in tumourigenesis including angiogenesis, enhanced cell migration, invasion and chemo-resistance. Although the molecular mechanisms driving some of these interactions are described (β1-integrins/BSG or CD44/BSG), further investigation is needed to determine whether all the putative functions attributed to BSG result from a real physical interaction with the companion molecule or to its metabolic effects.

## Targeting components of the MCT/BSG complexes: a new hope for anticancer therapy

### Targeting BSG

Due to the interdependency of MCT1/4 and BSG for functional expression of lactate transport, and also to the key role of this glycoprotein in cancer development, it seems evident to consider BSG as a promising therapeutic target in cancer. Thus, genetic silencing studies on BSG have reported inhibition of tumour growth and increased cell death in different cancer cell lines associated with reduced, angiogenesis, MMP secretion, invasiveness and chemo-resistance [164–166, 131, 167]. On the other hand, treatment of human head and neck squamous cell carcinoma with the anti-BSG monoclonal antibody (CNTO3899) was found to reduce proliferation and induce caspase-mediated apoptosis of cells ex vivo, and to impair tumour growth with increased radio-sensitivity in vivo [168, 169]. Monoclonal antibodies against BSG have also shown efficacy in treatment of hepatocellular carcinoma and hypervascular pancreatic tumours when administered alone or in combination with chemotherapy [170].

### Targeting MCTs

The relevance of targeting lactic acid efflux to develop an anticancer strategy was initiated by the pharmacological inhibition and genetic knockdown of MCTs. Several small molecules were first described to efficiently inhibit MCT1 transport [94, 92]. Among these, mainly α-cyano-4-hydroxycinnamate (CHC) has been used by several groups and has demonstrated promise in inhibiting MCTs as a cancer therapy without any apparent toxicity in vivo [171, 172, 92, 173, 137]. However, the possible use of this first generation of inhibitors in the clinic faced the problem of their lack of MCT specificity. Consequently, data from all these studies did not validate MCT as an anticancer target [94].

**Fig. 5:**
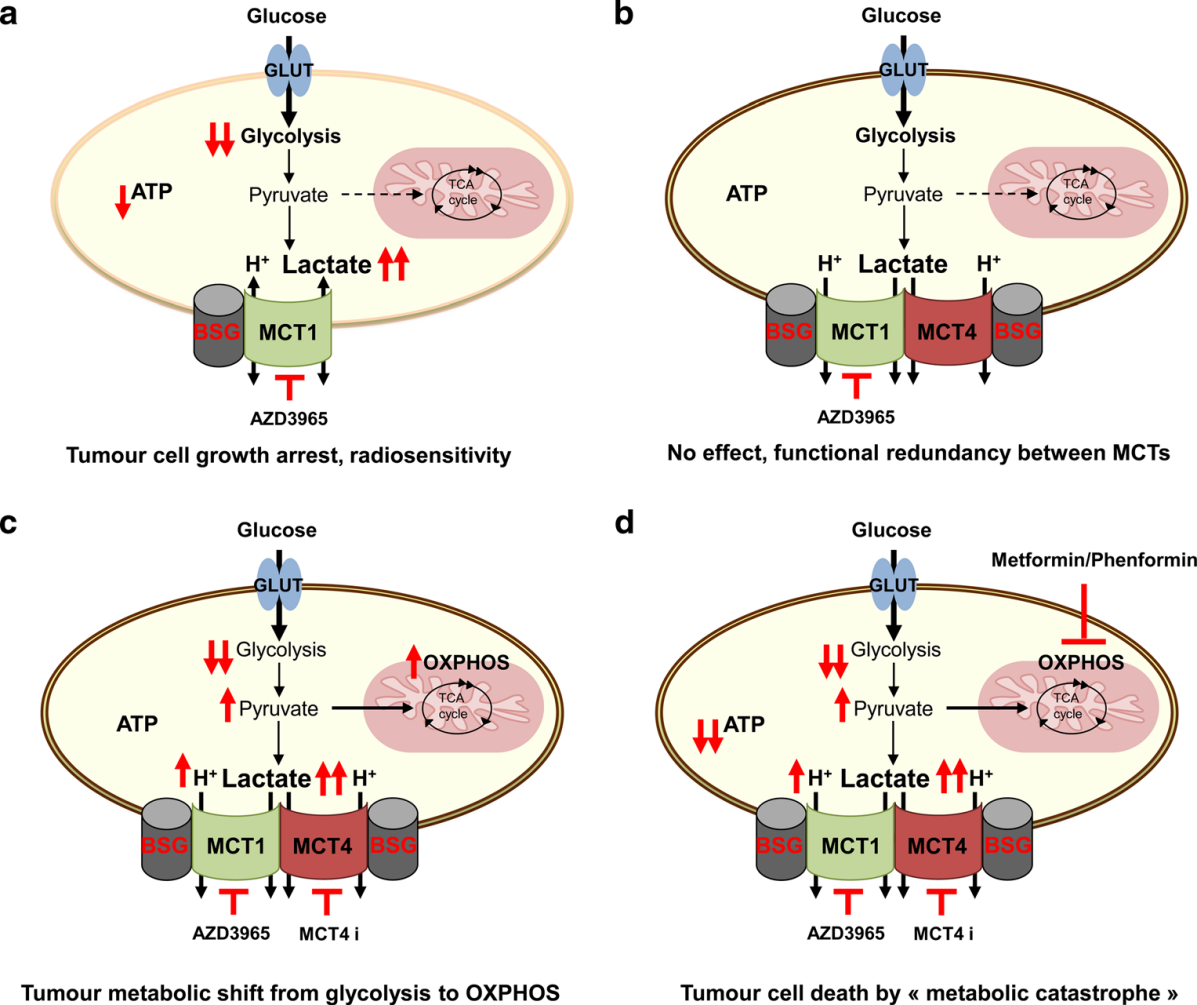
Efficiency of targeting lactate/H+ symporters for anticancer therapy. **a** Few oxidative cancer cells could use lactate to generate ATP, thus inhibition of monocarboxylate transporter 1 (MCT1) with AstraZeneca’s specific inhibitor AZD3965 results in growth arrest. Other type of cancer cells, glycolytic and expressing only MCT1, will be also sensitive to MCT1 inhibitor showing growth reduction, cell death and radiosensitivity. **b** Most of glycolytic cancer cells are expressing both MCT1 and MCT4. Due to functional redundancy between these two MCTs, AZD3965 will have no effect on hypoxic regions of the tumours. **c** Combined inhibition of MCT1 and MCT4 results in decreased glycolytic rate and severe growth arrest. However, increased intracellular lactic acid pool and subsequently increased intracellular pyruvate concentration, will fuel the tricarboxylic (TCA) cycle leading to metabolic shift from glycolysis towards OXPHOS. Therefore, tumour cells, although growing slowly, will survive by keeping physiological ATP pool and escape lactate export blockade. **d** Concomitant application of MCT inhibitors with metformin or phenformin, which inhibits OXPHOS, induces synthetic lethality resulting in “ATP crisis”. Consequently, rapid tumour cell death occurs due to “metabolic catastrophe.” Basigin (BSG); MCT4 inhibitor (MCT4 i)

Recently, AstraZeneca developed a new class of a highly specific and potent MCT1/MCT2 inhibitor (Ki values in the nmol/L range), named AR-C155858 [174] capable to increase intracellular pool of lactate [128]. Originally developed as an immunosuppressive drug that acts on T lymphocyte activation [175, 176], AR-C155858 has shown a striking impairment of both in vitro and in vivo growth of HRas-transformed fibroblasts, which established for the first time the relevance of targeting MCT1 in cancer [128]. Additionally, a second generation of more potent MCT1 inhibitors, AZD3965 (Km = 1.6 nmol/L), was recently reported to disrupt lactate efflux, glycolysis and glutathione synthesis of Burkitt lymphoma and MCF7 breast cancer cell lines, leading to cell death [13]. Other studies have also shown the anticancer effects of AZD3965 in small cell lung cancer (SCLC) and gastric cancer cells lines [177]. Treatment of tumours in vivo with the inhibitor induced an increase in the lactate concentration, reduced growth and enhanced radiation sensitivity [178] (Fig. 5a). AZD3965 is currently undergoing phase I/II clinical trials in the UK for patients with solid tumours, prostate cancer and diffuse large-cell B lymphoma.

Nevertheless, due to functional redundancy between MCT1 and MCT4, we and others have demonstrated that MCT1 inhibitors are inefficient in affecting growth and survival of highly glycolytic and hypoxic tumours (Fig. 5b). Indeed, ectopic expression of MCT4 in HRas-transformed fibroblasts rendered them insensitive to MCT1 inhibition and increased their tumour growth in vivo [128]. Moreover, knockdown or knockout of MCT4 in human colon adenocarcinoma cells made them sensitive to MCT1 pharmacological inhibition, and so impaired their proliferation in vitro and tumour growth in vivo [128, 14] (Fig. 5c). MCT4 silencing was also reported to decrease cancer cell migration, as MCT4 closely interacts with β1-integrins at the leading edge of migrating cells [179, 127]. Considering these data and the fact that most of highly aggressive tumours predominantly express the hypoxia-induced isoform MCT4, there is an absolute need to develop MCT4-specific inhibitors as a valuable anticancer therapy.

Recently, using a molecular model of MCT4, Nancolas et al. [180] reported structural differences between MCT1 and MCT4, and identified N147, R306 and S364 as key residues involved in MCT1 inhibitor (AR-C155858) binding and selectivity, which gives hope for development of selective small drugs inhibiting specifically MCT4. In the meantime, AstraZeneca succeeded in generating an MCT4 (AZ93) inhibitor that is likely selective and highly efficient in blocking growth of a wide range of cancer cells, but only when MCT1 gene is disrupted or MCT1 inhibited pharmacologically (Marchiq I, Critchlow S and Pouyssegur J, unpublished data).

Although MCT/BSG complex targeting therapies have shown great efficacy in several cancer cell lines, questions regarding their toxicity to normal tissues require further investigation. The wide spread expression of MCTs and BSG, in addition to the large spectrum of their functions (cellular metabolism, pHi, angiogenesis, immune response) imply necessarily the emergence of possible side effects, specifically in MCT/BSG highly expressing organs, such as heart, skeletal muscle, eyes, colon and genital organs [107, 129]. AstraZeneca have already included possible alterations of some organs in the phase I clinical trial of AZD3965.

Ultimately, the problem of targeting cellular metabolism is the interconnection between all the pathways and the ability of cancer cells to switch from one phenotype to another in order to produce energy and sustain viability and slow growth, which raises the question of resistance to anticancer therapies and tumour recurrence. In the case of MCT/BSG inhibition, recent work shows that blocking both MCT1 and MCT4 in human colon adenocarcinoma, glioblastomas and non-small cell lung carcinoma cells causes a shift of their metabolism from glycolysis to OXPHOS, which sensitise them to biguanides, such as metformin and phenformin [134, 14]. Similar observations were reported for Raji lymphoma and breast cancer cells [13]. Concomitant inhibition of glycolysis (MCTs inhibition) and mitochondrial complex I (biguanides) induces “ATP crisis” or “metabolic catastrophe” leading to rapid tumour cell death and tumour growth collapse (Fig. 5d). Similar synthetic lethality was described for prostate and colon cancer cells, in which combined treatment with 2-deoxyglucose and metformin or phenformin alone resulted in extensive cell death [181, 182]. Moreover, Dorr et al. have recently reported an interesting model of synthetic lethality of therapy-induced senescence lymphomas treated with inhibitors of glycolysis or autophagy [183]. However, such strategies now require further pharmacological evaluation in immune competent and genetically engineered mouse tumour models. First, an acceptable therapeutic window of combined MCT1/MCT4 inhibitors needs to be determined. Then, a second acute treatment of 1 to 3 days with phenformin should be tested for toxicity and tumour eradication. Considering the potency and the selectivity of the two MCT1 and MCT4 inhibitors developed by AstraZeneca and the large spectrum of growth arrest obtained in all of the human tumour cell lines analysed, we are very optimistic for future clinical development of these new drugs.

## Conclusion

Metabolic alteration has recently been recognised as an emerging “Hallmark” of cancer. The glycolytic switch in cancer cells not only provides cells with energy and biomolecules but also contributes to cell-cell communication. Recent evidence supports the notion of metabolic symbiosis within tumours, in which cancer and stromal cells use lactate as a metabolic fuel and signalling molecule, mimicking thus pre-existing and high-performance physiological mechanisms [184]. However, unlike the neurone-astrocyte shuttle or muscle fibre-red blood cells shuttle, the lactate circuit in tumours remains still poorly understood, mainly due to the complexity of the tumour microenvironment and interconnections between individual cellular subtypes. Thus, additional preclinical studies are needed to confirm and reinforce the available data. In addition, although, issues concerning when and where lactate exchanges occur are unresolved, regulators implicated in its handling are being characterised. Therefore, MCTs offer a great potential for developing new anticancer therapies based on disruption of lactate and pHi homeostasis. Transfer of MCT1 inhibitors developed by AstraZeneca from bench to clinical trials constitutes a first step in a long process validating the success of this strategy.
